# Transportan 10 improves the pharmacokinetics and pharmacodynamics of vancomycin

**DOI:** 10.1038/s41598-019-40103-w

**Published:** 2019-03-01

**Authors:** Jarosław Ruczyński, Izabela Rusiecka, Katarzyna Turecka, Agnieszka Kozłowska, Magdalena Alenowicz, Iwona Gągało, Anna Kawiak, Piotr Rekowski, Krzysztof Waleron, Ivan Kocić

**Affiliations:** 1 Faculty of Chemistry, University of Gdansk, Wita Stwosza 63, 80-308 Gdansk, Poland; 2 Department of Pharmacology, Medical University of Gdansk, Debowa 23, 80-204 Gdansk, Poland; 3 Department of Pharmaceutical Microbiology, Faculty of Pharmacy, Medical University of Gdansk, Hallera 107, 80-416 Gdansk, Poland; 4 Department of Biotechnology, Intercollegiate Faculty of Biotechnology, University of Gdansk and Medical University of Gdansk, Abrahama 58, 80-307 Gdansk, Poland

**Keywords:** Drug delivery, Drug delivery, Pharmacology, Antimicrobials, Drug development

## Abstract

In the presented study, transportan 10 (TP10), an amphipathic cell penetrating peptide (CPP) with high translocation activity, was conjugated with vancomycin (Van), which is known for poor access to the intracellular bacteria and the brain. The antibacterial activity of the conjugates was tested on selected clinical strains of methicillin-resistant *Staphylococcus aureus* (MRSA) and *Enterococcus sp*. It turned out that all of them had superior antimicrobial activity in comparison to that of free Van, which became visible particularly against clinical MRSA strains. Furthermore, one of the conjugates was tested against MRSA - infected human cells. With respect to them, this compound showed high bactericidal activity. Next, the same conjugate was screened for its capacity to cross the blood brain barrier (BBB). Therefore, qualitative and quantitative analyses of the conjugate’s presence in the mouse brain slices were carried out after its *iv* administration. They indicated the conjugate’s presence in the brain in amount >200 times bigger than that of Van. The conjugates were safe with respect to erythrocyte toxicity (erythrocyte lysis assay). Van in the form of a conjugate with TP10 acquires superior pharmacodynamic and pharmacokinetic.

## Introduction

Vancomycin (Van), as a member of glycopeptide antibiotics^1^, possesses bactericidal activity against Gram-positive aerobes and anaerobes. This action is due to its binding to the D-alanyl-D-alanine (D-Ala-D-Ala) C-terminus of the pentapeptide, which has the effect of blocking the addition of late precursors by transglicolization to the nascent peptidoglycan (PG) chain. Van is often used to treat life-threatening infections induced by multidrug resistant (MDR) bacteria such as *S*. *aureus*, *Enterococcus spp*. and *C*. *difficile*. At present, these microorganisms are a leading cause of community-acquired infections that result in high morbidity and death rates. Among them, severe MRSA infections are true medical emergencies and their treatment presents a major challenge to the medical community^2^.

Despite Van’s therapeutic relevance there are also shortcomings to its use. Of great concern, is the emergence of resistance among methicillin-resistant *Staphylococcus aureus* (MRSA) and enterococcal strains. This phenomenon is due to the modification of Van’s binding target (replacement of C-terminal D-Ala residue by D-lactate or D-serine) which indicates low affinity to the antibiotic. At first, resistance of enterococci to Van was discovered (1980s), next within MRSA strains (about 2000s). It turned out that a part of vancomycin-resistant enterococci conjugative plasmid may be transferred (*vanA* gene operon encoded on transposon *Tn1546*) to MRSA during discrete conjugation events, and this leads to complete Van resistance of *S*. *aureus* (VRSA). Fortunately, VRSA strains emerge rarely in hospital and community settings (1 in Europe, 14 in the U.S.). A much higher prevalence concerns h-VISA (heterogenous vancomycin intermediate *S*. *aureus*) or VISA which poses a significant threat because these bacteria produce persistent infection requiring hospitalization, prolonged vancomycin medication with a considerable risk of treatment failure^3^.

Another important limitation of Van results from its chemical features. Being a hydrophilic molecule, this antibiotic has weak access to the cell interior or specific regions of the body such as the brain^4–8^.

To tackle Van’s limitations, versatile strategies and novel alternative procedures have been undertaken. Successful approaches in this field have focused on among others:

1. Multiplication of binding sites in Van molecule – a multivalent polymer of Van^9^,
2. Improving Van’s binding affinity to the modified target of the resistant bacteria - conjugates of Van with carbohydrates, Van-capped Au or Ag nanoparticles^10–12^,
3. Enhancing the transport of Van to the intracellular compartment of the infected cells and the bacteria themselves - Van as a component of diverse delivery platforms as liposomes, nanoparticles, blend of polymers, cell-penetrating peptides (CPPs), composite beds of chitosan nanoparticles loaded with poly (trimethylene carbonate)^8,13–16^,
4. Increasing recognition and selective trapping Gram+ pathogens - Van-modified mesoporous silica or magnetic nanoparticles^17,18^,
5. Adding a new site of binding which may appear within the same target - hybridization of Van with an antimicrobial peptide like nisin or, in an additional one - synthetic modifications of Van involving hydrophobic appendages^19,20^.

Most of the above-presented strategies (also that with CPPs) concentrated on obtaining constructs with Van and compounds which lacked antimicrobial activity *per se*. An approach involving conjugation of this antibiotic with a CPP with a documented bactericidal action (TP10 is one of the few with such propensity) would meet better the requirements for an appropriate therapeutic candidate. Such a strategy has been used in this study, and therefore, Van was covalently conjugated with TP10. This formulation approach, despite its pros and cons, shows crucial superiority over physical complexation (simple bulk-mixing) since covalent conjugation of a CPP to its cargo results in a well-defined molecule^21,22^. This enables designing a conjugate which will constitute a novel therapeutic agent with a predictable chemical structure and activity.

TP10, being a representative of cationic CPPs, is a 21-residue chimeric and primary amphipathic construct comprising the N-terminal part of the neuropeptide galanin being linked to the full lengths wasp venom peptide mastoparan^23^. It contains four basic lysine residues and can attract up to 5 positive charges at physiologic pH^24^.This peptide is known not only for transporting versatile cargos across the cell membranes but also for its antimicrobial activity *per se*^25–27^. The mechanisms by which TP10 penetrates cell membranes are believed to involve both endocytosis and direct translocation however, they are still widely investigated and discussed^22,28,29^. With respect to its antimicrobial action it was demonstrated that this CPP is bactericidal, through its ability to disturb the integrity of the bacterial membrane and bind to genomic DNA. TP10 kills many Gram-positive and negative bacteria including MDR (multi-drug resistance) clinical strains^25,30^ and prevents inflammatory responses upon infection^30^.

It is known that cargo coupling position is an important aspect to consider when designing conjugates, particularly in the case of TP10 because it determines the activity of the conjugate as well as its uptake pattern, fragmentation and cytotoxicity^24,31–33^. Taking into account the relationship between chemical structure and pharmacologic properties, four conjugates were synthesized: Van-PEG_3_-TP10, Van-PEG_4_-TP10, TP10-Ala(PEG_4_-Van), [Lys^7^(PEG_4_-Van)]TP10. Van was attached to the N- or C-terminal ends or to Lys^7^ of the TP10 molecule. Thanks to this strategy, the most active conjugates, i.e those with improved pharmacodynamic and/or pharmacokinetic qualities in comparison to those of Van could be determined.

With respect to pharmacodynamics, all conjugates were tested on selected clinical strains of MRSA and *Enterococcus spp*. (*E*. *faecium* and *E*. *faecalis*) in order to determine MIC scores and identify MIC breakpoints. Moreover, MICs for a mixture of Van with TP10 were found out in order to establish the potential interaction between its components.

Since MRSA is dwelling inside the host cells, it was interesting to establish whether the compounds in question gained access to the intracellular compartment. Therefore, one of the conjugates, [Lys^7^(PEG_4_-Van)]TP10 was assessed by using an intracellular antimicrobial assay. This conjugate was chosen as it is known that the connection of TP10 *via* the side chain of Lys^7^ to the cargo guarantees optimal intracellular delivery^31,32^.

Pharmacokinetics, in turn, included *in vivo* experiments carried out with the same conjugate i.e. [Lys^7^(PEG_4_-Van)]TP10. The purpose of them was to determine whether this conjugate is able to cross the BBB. It is well documented that this barrier impedes the transport of therapeutic agents to the brain, and thereby limiting the treatment of many cerebral diseases. Among these agents are small and large molecules, including the compound in question, i.e. Van.

Therefore, if the covalent conjugation used in this study truly promotes the passage of therapeutic agents into the CNS, this finding will be of great importance from the clinical point of view. To confirm it, the presence of the above-mentioned conjugate was analyzed in the mouse brain after its peripheral administration by using qualitative (fluorescent microscopy) and quantitative (LC/MS) methods.

Additionally, the quantitative experimental procedure was repeated for a simple bulk-mixture of Van with TP10.

If TP10 is regarded as a possible future candidate for drug delivery, much consideration should be given to its potential toxicity. It must be kept at a minimum if this CPP is to be used as a drug delivery vector. There are data indicating that the toxicity of CPPs depends heavily on peptide concentration, cargo molecule and coupling strategy^31^. This study, however, focuses mainly on pharmacokinetic and pharmacodynamic aspects of TP10 because a precise analysis of its toxicity on different human cell lines has been already presented in a former study^34^. Thus, only two conjugates with different linkers, i.e. Van-PEG_3_-TP10 and Van-PEG_4_-TP10 were investigated with respect to their erythrocyte toxicity (erythrocyte lysis assay). This kind of experiment was undertaken to determine whether mastoparan (a fragment of TP10) known for strong hemolytic activity^35^, will have an impact on this toxicity profile of the conjugates.

## Material and Methods

### Reagents

All reagents and solvents (analytical, HPLC-grade or LC-MS grade) were purchased from Sigma-Aldrich Co (Poznań, Poland). All solutions were freshly prepared and the solvent was distilled deionized water (Milli-Q Millipore system, Bedford, USA). They were filtered with a 0.22 μm filter before usage. Fmoc protected l-amino acids used for peptide synthesis were obtained from Bachem AG (Bublendorf, Switzerland). Rink-Amide TentaGel S RAM resin (capacity 0.25 mmol/g) for the peptide synthesis was obtained from Rapp Polymere GmbH (Tuebingen, Germany). 15-azido-4,7,10,13-tetraoxapentadecanoic acid *N*-hydroxysuccinimidyl ester (N_3_-PEG_4_-NHS), 1-amine-11-azido-3,6,9-trioxaundecane (N_3_-PEG_3_-NH_2_) and 6-carboxyfluoresceine *N*-hydroxysuccinimidyl ester (Fl-NHS) were purchased from ChemPep Inc. (Wellington, USA).

### The sequence of TP10 and its conjugates with Van

The investigated compounds, TP10 and its conjugates with Van, as well as their amino acid sequence are presented in Table 1. Conjugate IV was used without and with coupling to fluorescein (IVa).

**Table 1:** The names of the compounds and their sequence.

	Compound name	Sequence
	TP10	AGYLLGKINLKALAALAKKIL-*amide*
I	Van-PEG_3_-TP10	Van-NH-PEG_3_-Tra(1,4)-C(O)-AGYLLGKINLKALAALAKKIL-*amide*
II	Van-PEG_4_-TP10	Van-C(O)-PEG_4_-Tra(1,4)-C(O)-AGYLLGKINLKALAALAKKIL-*amide*
III	TP10-Ala(PEG_4_-Van)	AGYLLGKINLKALAALAKKIL-Ala(Tra(1,4)-PEG_4_-C(O)-Van)-*amide*
IV IVa	[Lys^7^(PEG_4_-Van)]TP10	AGYLLGK^7^(C(O)-Tra(1,4)-PEG_4_-C(O)-Van)INLKALAALAKKIL-*amide*
	*Fl*-[Lys^7^(PEG_4_-Van)]TP10	*Fl*-AGYLLGK^7^(C(O)-Tra(1,4)-PEG_4_-C(O)-Van)INLKALAALAKKIL-*amide*

*Fl* – fluorescein, PEG_4_ – 4,7,10,13-tetraoxapentadecane linker, PEG_3_ – 3,6,9-trioxaundecane linker, Tra – 1,2,3-triazole ring.

### Chemical synthesis

#### Synthesis of TP10 and its analogues

TP10 and its analogues were synthesized according to the standard procedure of the solid phase peptide synthesis (SPPS) by using an automatic peptide synthesizer (Quartet, Protein Technologies Inc) with TentaGel S RAM resin (loading of amino groups − 0.25 mmol/g)^36,37^. Fmoc-protected amino acids were assembled as active derivatives in a 3-fold molar excess of *O*-(benzotriazole-1-yl)-1,1,3,3-tetramethyluronium tetrafluoroborate (TBTU) with addition of *N*-hydroxybenzotriazole (HOBt) and *N*-methylmorpholine (NMM) (1:1:2) in the *N*,*N*-dimethylformoamide (DMF) solution for 2 × 30 min. Removal of the fluorenyl-9-methoxycarbonyl (Fmoc) group was carried out with 20% piperidine/DMF in 2 cycles (2 × 3.5 min). Additionally, a hydrazine-labile 1-[4,4-dimethyl-2,6-dioxocyclohex-1-ylidene)-3-methylbutyl (ivDde) group was used to protect the *ε*-amino function group at Lys^7^ residue instead of the standard acid-labile *tert*-butyloxycarbonyl (Boc) group (the conjugate **IV** and **IVa** synthesis). As hydrazine removes the Fmoc group, the *N*-terminal of Ala residue (in conjugate **IV**) was coupled as Boc-protected amino acid. In conjugate **IVa** synthesis, 6-carboxyfluorescein (*Fl*) was coupled to the *N*-terminal amino group using a 3-fold molar excess of 6-carboxyfluorescein *N*-hydroxysuccinimidyl ester (Fl-NHS) with the addition of *N*,*N*-diisopropylethylamine (DIPEA) (1:1) in DMF for 1.5 h.

#### Modifications of TP10 backbone with alkyne group

In the case of conjugate **I** and **II** synthesis, the *N*-terminal Fmoc group was removed with 20% piperidine/DMF (2 × 5 min), and the propiolate group (Prop) was attached to the *N*-terminal amino group by using a 10-fold molar excess of propiolic anhydride in DMF for 1.5 h. Propiolic anhydride was obtained by mixing *N*,*N*′-diisopropylcarbodiimide (DIC) with propiolic acid (1:2) in dichloromethane (DCM). The mixture was stored at 0 °C for 10 min. The precipitate was filtered off, and after evaporation of DCM the residue was dissolved in 5 ml of DMF and added to the reaction vessel containing peptidyl-resin. However, the alkyne functionalization for conjugate **III** was obtained by attachment of the Fmoc-l-propargylglycine (Fmoc-Prg-OH) to the resin prior to the assembly of the TP10 sequence. In the case of conjugate **IV** and **IVa** synthesis, the ivDde group was removed with 10% hydrazine monohydrate/DMF (3 × 20 min) and the alkyne group was attached to the *ε*-amino function group of Lys^7^ residue by using a 10-fold molar excess of propiolic anhydride (as described above).

#### Cleavage of TP10 peptides from the resin

Immobilized peptides were cleaved from resins and deprotected with a mixture of trifluoroacetic acid (TFA), phenol, triisopropylsilane and water (88:5:2:5) for 2 h. The precipitation of the peptides from the reaction mixtures was obtained by using cold diethyl ether. Next, the precipitate was filtered, dissolved in water and lyophilized. The crude peptides were analyzed and purified by reverse-phase high-performance liquid chromatography (RP-HPLC). Finally, the identities of products were confirmed by MALDI-TOF mass spectrometry (Bruker Daltonics, model HCT Ultra) or ESI mass spectrometry (ABSciex, TripleTOF 5600+).

#### Modifications of Van structure

The azido functionalized Van derivatives were synthesized in solution. In the case of Van-PEG_4_-N_3_ derivative, 15-azido-4,7,10,13-tetraoxapentadecanoic acid *N*-hydroxysuccinimidyl ester (N_3_-PEG_4_-NHS) was coupled to vancomycin hydrochloride *via* the primary amino group in the sugar moiety. The reaction was performed in water solution with addition of DIPEA (1:1.5:2.5). The mixture was stirred at 4 °C for 30 min. However, Van-PEG_3_-N_3_ derivative was obtained by attachment of 1-amine-11-azido-3,6,9-trioxoundecane (N_3_-PEG_3_-NH_2_) to vancomycin hydrochloride *via* the carboxylic group in the aglycone moiety. The reaction was performed in DMF solution by using 2-(1*H*-7-azabenzo-triazole-1-yl)-1,1,3,3-tetramethylouronium hexafluorophosphate (HATU) with addition of DIPEA (1:0.8:1:2). The mixture was stirred at room temperature for 1 h. After this time, the reaction products were immediately separated by preparative RP-HPLC. Eluates were fractioned and analyzed by analytical RP-HPLC. Fractions of Van-PEG_4_-N_3_ or Van-PEG_3_-N_3_ with the purity >98% were collected and lyophilized. Finally, the identities of products were confirmed by MALDI-TOF or ESI mass spectrometry.

#### Synthesis of Van-TP10 conjugates

Conjugates of TP10 with Van were obtained by using “click chemistry” – the Cu(I)-catalyzed specific 1,3-dipolar Huisgen’s cycloaddition reaction^37^. The reactions of the alkyne functionalized TP10 analogues (0.8 μM each time) with 0.4 μM of azido functionalized Van-PEG_3_-N_3_ (conjugate **I**) or Van-PEG_4_-N_3_ (conjugates **II–IV**) were carried out in 1.5 ml of water/*tert*-butanol medium (1:1 v/v) in the presence of 8 µl of 0.1 M CuSO_4_ × H_2_O and 4 µl of freshly prepared solution of 0.5 M sodium ascorbate (2:1:2:5). The mixtures were stirred at room temperature for about 24 hours. After the 1,2,3-triazole forming reactions had been completed, the solvents were evaporated and the products lyophilized. Crude conjugates thus obtained were purified by preparative or semi-preparative RP-HPLC. Their purity was checked by analytical RP-HPLC (with the use of various gradients of acetonitrile and water) and established at the level >98%. The correctness of the molecular mass of the tested compounds was confirmed by ESI or MALDI-TOF mass spectrometry. Table 2 summarizes the calculated and experimental molecular masses of the conjugates obtained as well as the yields of the conjugation reactions.

**Table 2:** Comparison of the molecular masses of the conjugates and the yields of the conjugation reactions.

	Compound name	Molecular mass		Yield** [%]
		Calculated	Experimental	
I	Van-PEG_3_-TP10	3883.27	1294.36 [M + 3 H]^3+^, 971.03 [M + 4 H]^4+^, 777.04 [M + 5 H]^5+^, 647.97 [M + 6 H]^6+^	47
II	Van-PEG_4_-TP10	3956.32	1318.40 [M + 3 H]^3+^, 989.05 [M + 4 H]^4+^, 791.45 [M + 5 H]^5+^, 659.87 [M + 6 H]^6+^	85
III	TP10-Ala(PEG_4_-Van)	4001.40	3999.60* [M + H]^+^	76
IV	[Lys^7^(PEG_4_-Van)]TP10	3957.30	3956.80* [M + H]^+^	58
IVa	*Fl*-[Lys^7^(PEG_4_-Van)]TP10	4316.60	4317.10* [M + H]^+^	24

^*^Obtained from MALDI-TOF mass spectrometry.

^**^Only fractions with HPLC purity greater than 98% were considered.

#### RP-HPLC analysis and purification

Purifications of synthesized products were performed on a Reprosil 100 C18 column (Dr. Maisch GmbH, 40 × 250 mm, 10 µm particle size, flow rate 25 mL/min) or Reprosil 100 C18-XBD column (Dr. Maisch GmbH, 20 × 250 mm, 10 µm particle size, flow rate 10 mL/min) by using a SpotPrep (Armen) system and several gradient methods. The mobile phase consisted of 0.08% TFA in acetonitrile (ACN) (solvent A) and 0.1% TFA in water (solvent B). The column was maintained at ambient temperature. The eluted solution was monitored with an UV detector at 220 and 254 nm. Eluates were fractioned and analyzed by analytical RP-HPLC.

Analytical separations were performed on a Kinetex XB-C18 column (Phenomenex, 4.6 × 150 mm, 5 µm particle size, flow rate was 1 mL/min) using a Shimadzu Prominence system and several gradient methods. The mobile phase, ambient temperature and the parameters of UV monitoring were the same as those mentioned for the Reprosil columns.

**Figure 1:**
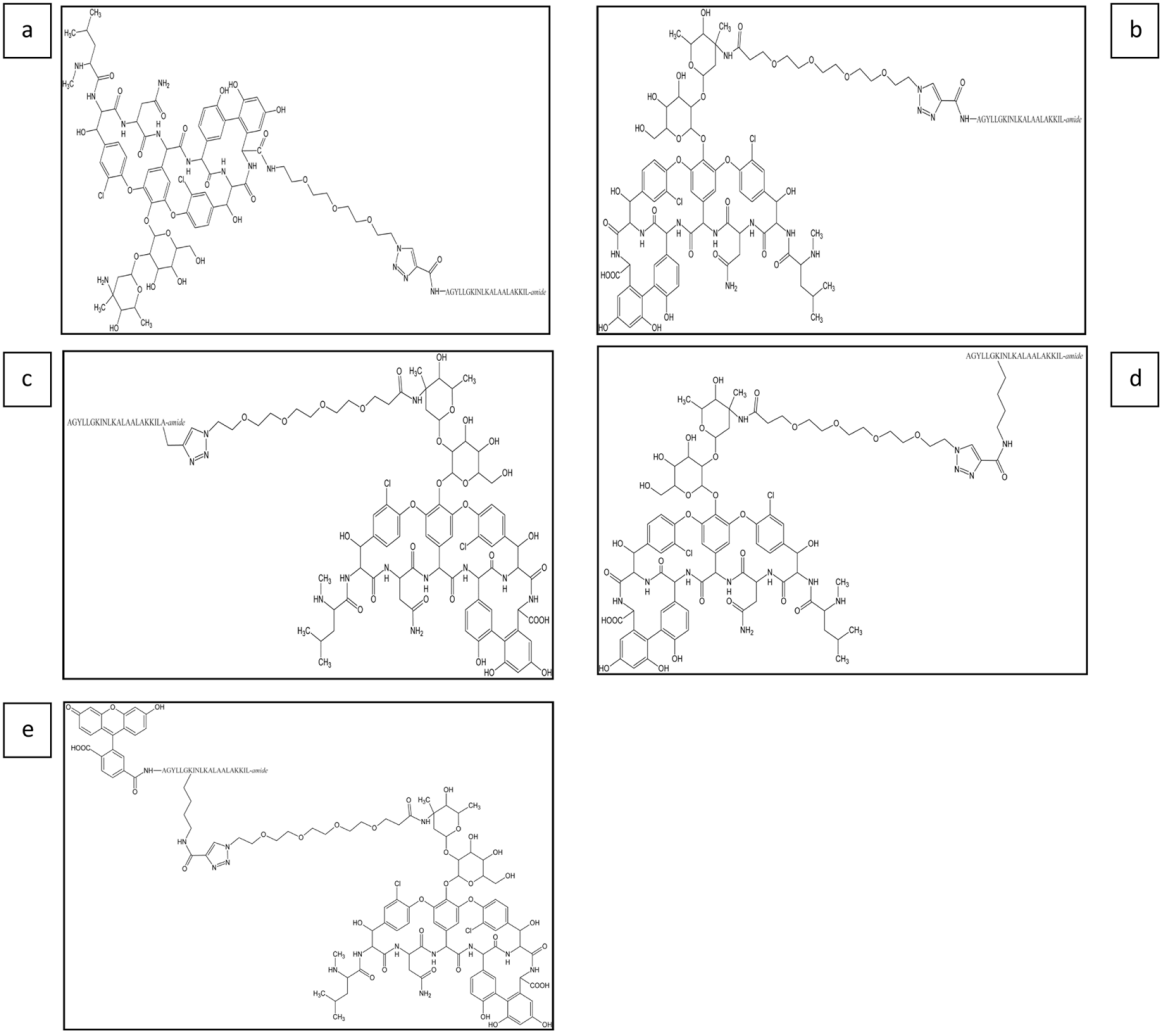
The chemical structure of the synthesized conjugates in question. The conjugates: (**a**) Van-PEG_3_-TP10 (conjugate I); (**b**) Van-PEG_4_-TP10 (conjugate II); (**c**) TP10-Ala(PEG_4_-Van) (conjugate III); (**d**) [Lys^7^(PEG_4_-Van)]TP10 (conjugate IV); (**e**) Fl[Lys^7^(PEG_4_-Van)]TP10 (conjugate V).

The chemical structure of the obtained conjugates are presented in Fig. 1.

### Preparation of Van + TP10 mixture

The mixture of TP10 + Van was prepared by mixing equal volumes of TP10 (10^−4^ M) and Van saline stock solutions. The final ratio of TP10/Van was 1:1.

### Antimicrobial activity

#### Van-TP10 conjugates

Three bacterial strains of MRSA (N315, 12673, 6347) and two of *Enterococcus spp*. (*E*. *faecium*, *E*. *faecalis*) were used. With respect to MRSA, N315 represented a reference Van-susceptible strain (GenBank GCA_00000645.1) isolated from a Japanese patient while 12673 (Van-intermediately susceptible) and 6347 (a h-VISA which appears at frequency of <10^−5^ to 10^−6^ is composed of two subpopulations of bacteria: one susceptible and one intermediately resistant)^38^ were clinical strains obtained from patients of The Clinical University Centre of Gdańsk (UCK). Both enterococcal strains were also of clinical origin (UCK) and they showed resistance to Van. The clinical strain isolates originated from the collection of the Department of Pharmaceutical Technology and Biochemistry, Faculty of Chemistry, Gdańsk University of Technology.

The strains were grown in Mueller-Hinton broth (MH cation-adjusted, Becton Dickinson) or Brain Heart Infusion broth (BHI, Becton Dickinson), respectively. The cultivation was carried out in an aerobic atmosphere at 37 °C for 24 hours and, afterwards, the bacterial cultures were diluted in geometric progression with a proper broth. Next, 100 µl of each dilution was inoculated in an agar plate and incubated at 37 °C for 24 h. The next procedures included determination of Colony Forming Units (CFU)/ml and minimal inhibitory concentration (MIC) of the compounds in question. The latter procedure was carried out according to the microbroth dilution technique using 96-well plates^39^. The MICs were evaluated by using the metabolic assay based on the resazurin dye. The assay was performed in triplicates.

#### The determination of an interaction between Van and TP10

In order to determine the possible interaction between Van (A) and TP10 (B), the fractional inhibitory concentrations (FICs) for both compounds were calculated as follows: FIC_A_ = [MIC_A(with B)_]/[MIC_A_], FIC_B_ = [MIC_B(with A)_]/[MIC_B_]. To find out MIC_A(with B)_ and MIC_B(with A)_ a checkerboard assay was carried out in 96-well microtiter plates (each test was performed in triplicates). The concentration range of Van and TP10 was from 12.5 to 0.012 µM. Each well in a 96-well plate was inoculated with 100 µL of bacterial inoculum of 1 × 10^5^ CFU/mL. For this purpose only bacterial strains of MRSA (N315, 12673 and 6347) were used. Next, the plates were incubated at 37 °C for 24 h. After incubation, MIC_Van+TP10_ and MIC_TP10+Van_ were read at MIC of TP10 (6.3 μM for MRSA N315, 12673 and 12.5 μM for MRSA 6347) or MIC of Van (2.0 μM for MRSA N315 or 4.1 μM for MRSA 6347 or 12673), respectively. Afterwards, FIC values for Van and TP10 were calculated in the following way: FIC_Van_ = MIC_Van+TP10_ divided by MIC of Van alone and FIC_TP10_ = MIC_TP10+Van_ divided by MIC of TP10 alone.

FIC index (FICI) is the sum of FIC_A_ and FIC_B_. The interpretation of the calculated FICIs is in accordance to the following principle: FICI ≤ 0.5 synergy; FICI > 0.5 and ≤4 indifference (no interaction); FICI > 4, antagonism^40^.

### Antimicrobial activity of [Lys^7^(PEG_4_-Van)]TP10 against intracellular MRSA strains

#### Cell Culture

HEK293 (human embryonic kidney) cells were purchased form Cell Line Services (Germany). The cells were cultured in Dulbecco’s Modified Eagle’s (DMEM) medium with 4.5 g/l glucose (Sigma-Aldrich, Germany), supplemented with 10% fetal bovine serum (FBS, Sigma-Aldrich, Germany) and 1% PEN/STREP (Sigma-Aldrich, Germany). Cells were maintained in a 5% CO_2_ incubator at 37 °C. The original lines were checked for the presence of mycoplasma.

#### Infection assay

HEK293 cells were seeded at a density of 1.5 × 10^5^ cells/well in 24-well plates in an antibiotic-free medium (DMEM + 10% FBS) and were allowed to adhere overnight. Next, the medium was removed and overnight culture of *S*. *aureus* strain (12673) suspended in DMEM + 1% FBS (invasive medium) at a ratio of bacteria/cells 25:1 was added. To enable bacterial invasion, the cells were incubated with bacterial suspensions (37 °C, 5% CO_2_) for 2 h.

After this period of time extracellular bacteria were eliminated by a 30 min incubation with 20 U/ml lysostaphin. After removal of these bacteria, DMEM + 10% FBS was added again and the cells were treated with Van, TP10 or [Lys^7^(PEG_4_-Van)]TP10 at the final concentrations of 25 µM for 24 h. The lysis of human cells was carried out with 0.2% Trition-X-100 (Sigma-Aldrich, Germany). The obtained cell lysates were serially diluted in PBS and plated on agar plates and CFU/ml was determined.

The results are presented as the percentage of *S*. *aureus* intracellular survival after treatment in comparison to that of non-treated cells (control). The assay was performed in triplicates.

### Estimation of BBB penetrating ability of Fl-[Lys^7^(PEG_4_-Van)]TP10 (qualitative method)

*In vivo* experiments were performed on BALB/c male mice weighing between 20 and 30 g (the mouse model is widely used for estimation of drug BBB penetration). The animals (obtained from TAZD-CBU) were kept in a 12-hour day and night cycle at room temperature (20–22 °C), humidity 55–56% and fed with a standard diet and water *ad libitum* for at least 1 week before the experiment. Body temperature and weight were recorded on a regular daily schedule. It is confirmed that all procedures were carried out according to the guidelines outlined by the European Community Council Directive 2010/63/EEC, and they were approved by the local Ethical Committee Resolution 26/2014 (Bydgoszcz, Poland).

Fl-[Lys^7^(PEG_4_-Van)]TP10 was administered to the mouse (n = 5) tail vein at a dose of 60 mg/kg in a volume of 100 μl of saline. The dose of the conjugate (60 mg/kg) relates to that of Van, which was established on the mouse model by others^41,42^.

After 2 hours, the mice were sacrificed (by isoflurane overdose) and their brains were immediately removed from the cranium and cooled to 4 °C. Five, 0.5 μm thick coronal cryo-sections of the brain were made starting from the frontal pole and moving to the occipital pole (HM 450 Sliding Microtome). The presence of the compound in question in the brain slices was visualized using a fluorescence microscope (Delta Optical IB-100, Delta Optical, Poland) at a fluorescence excitation and emission of 490 nm and 521 nm, respectively. The exposure time averaged 1 min., and the total magnification of microscopic images was 40x.

### Determination of the amounts of Van and [Lys^7^(PEG_4_-Van)]TP10 in the mouse brain by the use of LC/MS method (quantitative method)

The experiments in this section also involved the mouse model described above. The animals (ten mice per group) were treated with: (1) saline (controls), (2) Van, (3) TP10, (4) [Lys^7^(PEG_4_-Van)]TP10, (5) Van + TP10. The doses of Van and its conjugate or mixture were 60 mg/kg. Similarly to what was stated previously, the mice were sacrificed 2 hours after the administration of the compounds. Their brains were removed immediately from the cranium and weighed. Next, they were homogenized mechanically in the ice and frozen at −80 °C.

Each homogenized mouse brain was centrifuged at 15 000 × g for 10 min. The supernatant was diluted with deionised water (Millipore), filtered (PTFE 0.22 μm filter) and analyzed by UHPLC-ESI/MS with the use of Shimadzu Nexera X2 UHPCL chromatograph and Shimadzu LCMS-2020 mass detector. The chromatography was performed on the Phenomenex Kinetex XB-C18 (2.6 μm, 100 mm × 2.1 mm) column with several gradients of the mobile phase, which consisted of water/acetonitrile (with the addition of 0.1% formic acid and 0.01% trifluoroacetic acid) at a flow rate of 0.3 ml/min and temperature of 35 °C. The compounds in question were detected and analyzed by ESI-MS with the use of the selected ion monitoring mode (SIM):

$$\begin{array}{lll}m/z & = & 726.1\,{\rm{f}}{\rm{o}}{\rm{r}}\,{[{\rm{M}}+2{\rm{H}}]}^{2+}\,{\rm{a}}{\rm{n}}{\rm{d}}\,{\rm{m}}/{\rm{z}}=1450.7\,{\rm{f}}{\rm{o}}{\rm{r}}\,{[{\rm{M}}+{\rm{H}}]}^{+}\,{\rm{o}}{\rm{f}}\,{\rm{V}}{\rm{a}}{\rm{n}},\\ m/z & = & 989.8\,{\rm{f}}{\rm{o}}{\rm{r}}\,{[{\rm{M}}+4{\rm{H}}]}^{4+}\,{\rm{a}}{\rm{n}}{\rm{d}}\,{\rm{m}}/{\rm{z}}\\  & = & 1319.8\,{\rm{f}}{\rm{o}}{\rm{r}}\,{[{\rm{M}}+3{\rm{H}}]}^{3+}\,{\rm{o}}{\rm{f}}\,[{{\rm{L}}{\rm{y}}{\rm{s}}}^{7}{({\rm{P}}{\rm{E}}{\rm{G}}}_{4}-{\rm{V}}{\rm{a}}{\rm{n}})]{\rm{T}}{\rm{P}}10.\end{array}$$

The calibration curves obtained were linear in the range of 10–1000 ng/ml with linear correlation coefficient above 0.999.

### Erythrocyte lysis assay

Two of the Van conjugates i.e. Van-PEG_3_-TP10 (conjugate I) and Van-PEG_4_-TP10 (conjugate II) were taken for testing red blood cell toxicity. The protocol of the lysis assay is presented below.

To obtain sheep erythrocytes, 5 ml of fresh blood was centrifuged at 1000 × g for 10 min and washed 3 times with 0.9% NaCl. Cell suspension (final concentration of 10^8^ cells/ml) in a volume of 75 µl was added to each well of the 96-well microtiter plate and incubated at 37 °C in the presence of the tested compounds. The range of their concentrations was 100–0.049 µM (diluted in saline solution), which included that of MIC values for the tested strains of the bacteria.

To estimate the relative haemolytic potential of Van and its conjugates, appropriate controls i.e. 4% Triton X-100 (100% erythrocyte lysis) and saline solution (0% lysis) were used. Plates with samples were incubated at 37 °C for 1 hour and then centrifuged at 1000 ×  g for 10 min to separate the unlysed erythrocytes. Next, the supernatant was transferred to a new plate. The absorbance (A) was measured spectrophotometrically at 450 nm of wavelength. The experiments were performed in triplicates.

The haemolysis percentage was calculated according to the equation presented by Sharma *et al*.^43^. The percentage of haemolysis = [(A450nm of test compound treated sample-A450nm of buffer treated sample)/(A450nm of 4%TritonX-100 treated samples-A450nm of buffer treated sample)] × 100.

### Calculations and statistics

The MIC values (all tested conjugates and mixture), percentage of intracellular *S*. *aureus* survival, percentage of hemolysis (Van-PEG_3_-TP10, Van-PEG_4_-TP10) are expressed as a mean of at least three independent experiments, while LC/MS (Van, TP10, [Lys^7^(PEG_4_-Van)]TP10, Van + TP10) results are a mean of ten. All the experiments were conducted in triplicates.

On this basis, standard deviation (SD) was calculated. A one-way ANOVA test for statistical significance calculation (p < 0.05) was performed using the GraphPad Prism version 5.0 data analysis software system (GraphPad Software, San Diego California USA, www.graphpad.com). All results obtained for the conjugates were compared with those after Van treatment.

The fluorescent microscopy data is included from the experiments (each repeated five times) and two representatives of brain slice images are presented in the result section.

## Results

### The antibacterial activity of Van-TP10 conjugates

Due to the considerable differences between the molecular mass of the compounds in question, all MIC values are expressed both in μM and μg/ml (Table 3). Nevertheless, in order to demonstrate their action more adequately, molar concentrations (μM) were chosen for the presentation of the MIC values of the tested compounds as they correlate with the number of the reactive molecules. Table 4 presents the MIC breakpoints (μM) of Van for MRSA and *Enterococcus spp*.

**Table 3:** Antimicrobial activity of Van-TP10 conjugates.

The bacterial strain	MIC in μM and (μg/ml)					
	Van-HCl	TP10-*amide*	Van-PEG_3_-TP10-*amide*	Van-PEG_4_-TP10-*amide*	TP10-Ala(PEG_4_-Van)-*amide*	[Lys^7^(PEG_4_-Van)]TP10-*amide*
			I	II	III	IV
*S*. *aureus* MRSA N315^a^	2.0 ± 0.5 (3.0 ± 0.7)	6.3 ± 1.3 (13.6 ± 2.8)	1.6 ± 0.9 (6.1 ± 2.5)	1.6 ± 0.8 (6.3 ± 2.6)	12.5* ± 3.2 (50.0 ± 12.9)	6.3* ± 1.6 (24.7 ± 6.4)
*S*. *aureus* MRSA 12673^b^	4.1 ± 1.1 (6.1 ± 1.6)	6.3 ± 1.6 (13.6 ± 3.5)	1.6* ± 0.9 (6.1 ± 3.1)	1.6* ± 0.7 (6.3 ± 2.6)	0.8* ± 0.4 (3.1 ± 1.6)	0.8* ± 0.3 (3.1 ± 1.3)
*S*. *aureus* MRSA hetero-VISA 6347b^2^	4.1 ± 1.1 (6.1 ± 1.6)	12.5 ± 3.2 (27.3 ± 7.0)	1.6* ± 0.3 (6.1 ± 1.2)	3.1 ± 0.8 (12.3 ± 3.6)	1.6* ± 0.4 (6.4 ± 1.7)	3.1 ± 0.8 (12.3 ± 3.1)
*Enterococcus faecium* 3934825^b,c^	25.0 ± 6.5 (37.1 ± 7.6)	12.5 ± 3.4 (27.3 ± 10.9)	50.0* ± 10.2 (194.2 ± 39.6)	25.0 ± 5.1 (98.9 ± 20.2)	12.5* ± 3.2 (50.0 ± 12.9)	6.3* ± 1.7 (24.7 ± 6.8)
*Enterococcus faecalis* 3937158^b,d^	25.0 ± 6.5 (37.1 ± 9.6)	12.5 ± 2.2 (27.3 ± 7.0)	6.3* ± 1.6 (24.3 ± 6.3)	25.0 ± 6.5 (98.9 ± 21.8)	25.0 ± 5.1 (100.0 ± 20.4)	25.0 ± 6.9 (99.0 ± 25.5)
Molecular mass [g/mol]	1485.71	2181.91	3883.27	3956.32	3957.30	4316.60

^a^Reference strain.

^b^Clinical strain.

^c^Vancomycin resistant, linezolid sensitive.

^d^Cancomycin resistant, linezolid resistant.

*Statistically significant (p < 0.05) as compared to Van.

**Table 4:** Van breakpoints used for susceptibility determination of *S*. *aureus and Enterococcus spp*.

Bacterial strains	Susceptibility [μM]		
	S	I	R
*S. aureus*	≤1.38	2.69–5.38	>10.77
*Enterococcus spp*.	≤2.69	5.38–10.77	>21.54

S- sensitive; I – intermediate; R- resistant.

All MRSA strains tested in this study indicated intermediate susceptibility to Van treatment. The MIC values of the reference (N315) as well as clinical (6347 or 12673) strains were 2 μM and 4.1 μM, respectively. As could be expected, *Enterococcus spp*. were resistant to this antibiotic (MIC = 25 μM) (Table 3).

The MIC values for all bacterial strains after TP10 exposition were 6.3 or 12.5 μM, which according to Xie *et al*.^25^ reflects a satisfactory antibacterial effect. The lower value of the MIC range was noticed in the case of *S*. *aureus* reference (N315) and clinical (12673) strains.

All conjugates showed antibacterial action on the clinical MRSA strains, and the most susceptible was the one designated 12673. The MIC values after treatment of this strain with the tested conjugates were as follows: 0.8 μM (conjugate III and IV), 1.6 μM (conjugate I) and 1.6 μM (conjugate II). All of them differed significantly from MIC (4.1 μM) after treatment with Van. The most noticeable were MICs of conjugate III and IV, whose values, being 0.8 μM, were below the MIC breakpoint of Van for susceptible MRSA (≤1.38 μM).

In the case of the reference strain of MRSA an antibacterial effect was observed only after treatment with conjugate I (1.6 μM) and II (1.6 μM).

The action of Van conjugates against enterococcal strains was rather poor with the exception of conjugate I (MIC for *E*. *faecalis* = 6.3 μM) and conjugate IV (MIC for *E*. *faecium* = 6.3 μM). In both cases, MICs were shifted from the range of values characteristic of resistant eneterococci (MIC breakpoint for Van >21.54 μM) to that of the bacteria of intermediate susceptibility (MIC breakpoint for Van 5.38–10.77 μM).

### The interaction between Van and TP10

As Table 5 shows, the calculated FICIs for the tested MRSA strains are in the range of 0.7–0.9. These values indicate that neither synergy nor antagonism between Van and TP10 occurs (values of FICI between >0.5 ≤4 reflects indifference), even though a fold reduction of MIC values for both antimicrobials was observed. In the case of Van, an evident fold reduction (2.6) of its MIC in the presence of TP10 became visible after treatment of MRSA clinical strains. Notably, in the presence of Van the fold reduction of MICs of TP10 was in the range of 15.8–2.0 with the highest value concerning the reference bacterial strain.

**Table 5:** Antimicrobial activity of Van and TP10 in combination.

The bacterial strain	MIC in µM				Fold reduction of MIC of Van	Fold reduction of MIC of TP10	FICI^	Interpretation
	Van (A)	Van_(with TP10)_	TP10 (B)	TP10_(with Van)_				
	MIC_A_	MIC_A (with B)_	MIC_B_	MIC_B (with A)_				
*S*.*aureus* MRSA N315^a^	2.0	1.6	6.3	0.4	1.3	15.8	0.9	indifference
*S*.*aureus* MRSA 12673^b^	4.1	1.6	6.3	3.1	2.6	2.0	0.9	indifference
*S*.*aureus* MRSA hetero-VISA 6347^b^	4.1	1.6	12.5	3.1	2.6	3.9	0.7	indifference

^a^Reference strain.

^b^Clinical strain.

MIC_A (with B)_ was determined at a concentration of TP10 equal to 6.3 μM (MRSA N315 and 12673) or 12.5 μM (MRSA 6347).

MIC_B (with A)_ was determined at a concentration of Van equal to 2.0 μM (MRSA N315) or 4.1 μM (MRSA 12673 and 6347).

^The FICI data were interpreted using the following criteria: FICI ≤ 0.5 synergy; FICI > 0.5 and ≤4 indifference; FICI > 4 antagonism.

### Antimicrobial activity of [Lys^7^(PEG_4_-Van)]TP10 against intracellular MRSA 12673 strain

**Figure 2:**
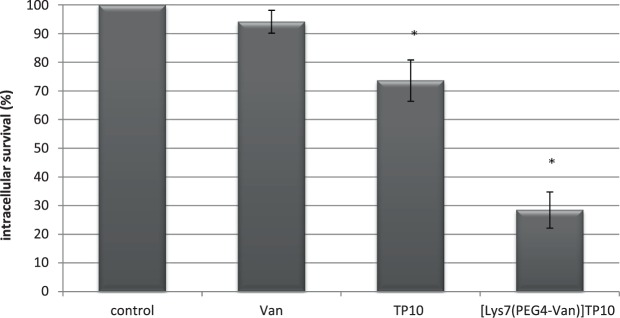
Antimicrobial activity of [Lys^7^(PEG_4_-Van)]TP10 against intracellular MRSA 12673 strain. ^*^Statistically significant (p < 0.05) as compared to control (no treatment) and Van.

The percentage survival of intracellular *S*. *aureus* after treatment with [Lys^7^(PEG_4_-Van)]TP10 is presented in Fig. 2. As could be predicted, the effect of Van on intracellular MRSA was weak. Almost 95% of the bacteria survived. TP10 produced a stronger effect with a bacterial survival of about 74% (p < 0.05). A prominent action on MRSA was observed due to [Lys^7^(PEG_4_-Van)]TP10. In the case of this treatment only approximately 29% of the bacterial population survived (p < 0.05). It should be added, that the described antibacterial effect of the conjugate was obtained after 32xMIC.

### Qualitative identification of Fl-[Lys^7^(PEG_4_-Van)]TP10 (conjugate IVa) in the brain

To evaluate the penetrating ability of Fl-[Lys^7^(PEG_4_-Van)]TP10 to cross the BBB the fluorescence microscopy assay was used.

**Figure 3:**
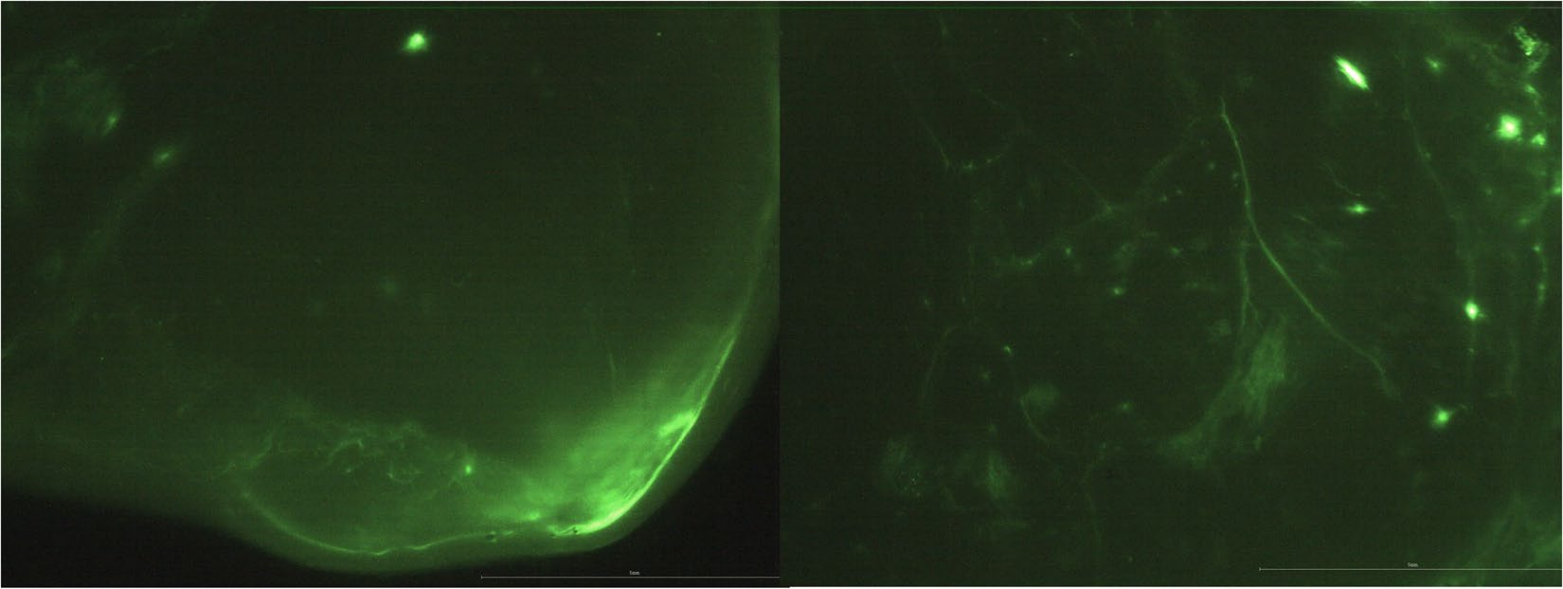
Fluorescent images of Fl-[Lys^7^(PEG_4_-Van)]TP10 distribution in mouse brain sections (4x objective). (**A**) Lower part of the right brain hemisphere with the olfactory tract. (**B**) Middle part of the right hemisphere with striatum. The images are a representative of three independent experiments. The scale bar reflects 5 mm in the brain images.

Figure 3A and B present the images of Fl-[Lys^7^(PEG_4_-Van)]TP10 in two different mouse brain slices obtained from distinct regions of the brain i.e. olfactory tract and striatum, respectively. This conjugate was chosen for qualitative analysis because among the tested conjugates it (as the only one) shifted the MIC values of clinical MRSA and *E*. *faecium* to those characteristic of susceptible and intermediate susceptibility, respectively.

The appearance of green illuminating color as is shown in the below presented figures, reflects the presence of the conjugate coupled with fluorescein in the brain and the degree of its concentration (lower/higher). The character of the dye distribution in both brain slices is different. In the case of Fig. 3A, the fluorescent dye is spread over a large area with an accumulation at the base of the brain (high concentration of Fl-[Lys^7^(PEG_4_-Van)]TP10), while in Fig. 3B, the most characteristic feature are green illuminating spots, which are of versatile size and brightness. What is more, the dye in the latter image is visible in the cerebral tissue as well as the blood vessels.

### Quantitative identification of [Lys^7^(PEG_4_-Van)]TP10 (conjugate IV)

Table 6 presents brain concentrations of Van, TP10, conjugate IV (the same one which was estimated qualitatively in the brain) and mixture of Van with TP10 (Van + TP10) after *iv* treatment of mice. As could be expected, Van itself poorly penetrated the BBB (11 nM) and TP10 at all. On the other hand, the conjugate IV gained evident access to the brain (2611 nM). The cerebral amount of the conjugate was much higher (more than 200) in comparison to that of Van. In contrast to the conjugate, the brain tissue was inaccessible for the mixture (Van + TP10).

**Table 6:** Concentrations of [Lys^7^(PEG_4_-Van)]TP10, Van and TP10 in mouse brain homogenates.

Treatment (*iv*)	Brain concentrations [nM] as (Mean ± SD)		
	Van	TP10	[Lys^7^ (PEG_4_-Van)]TP10
saline	ND		ND
Van	11 ± 2		ND
TP10		ND	
[Lys^7^(PEG_4_-Van)]TP10	ND		2611* ± 120
Van + TP10	ND	ND	

ND – not detected.

^*^Statistically significant (p < 0.05) as compared to Van.

### Erythrocyte toxicity

In the erythrocyte lysis assay, Van did not show hemolytic activity at the concentration range used in this study. Also, a lack of hemolysis was noted if erythrocytes were exposed to Van-PEG_3_-TP10 or Van-PEG_4_-TP10 at the concentrations reflecting their MIC values for the susceptible or intermediately susceptible strains (6.25–0.78 μM).

**Figure 4:**
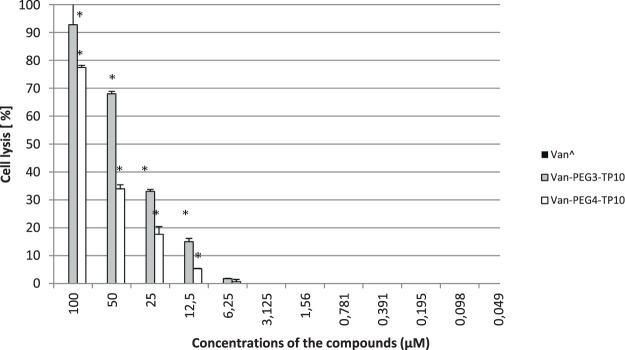
The hemolytic activity of Van, Van-PEG_3_-TP10 and Van-PEG_4_-TP10 in the concentration range of 100–0.049 µM measured spectrophotometrically at 450 nm. ^^^Van − 0% haemolysis throughout the whole concentration range. ^*^Statistically significant (p < 0.05) as compared to Van.

A progressive visible hemolysis due to the conjugates occurred starting from the concentrations 12.5 µM (15% and 5% cell lysis for Van-PEG_3_-TP10 or Van-PEG_4_-TP10, respectively). Among them, conjugate Van-PEG_3_-TP10 indicated always a stronger hemolytic activity. At the concentration of 100 µM, cell lysis achieved 93% for Van-PEG_3_-TP10 and 77% for conjugate Van-PEG_4_-TP10 (Fig. 4).

## Discussion

As the study indicated, all of the conjugates in question possessed a more evident antibacterial activity against clinical strains of MRSA in comparison to that observed after separate incubation with Van or TP10. The extent to which the antibacterial effect was intensified depended on the strain and the site of Van substitution in the TP10 molecule. For example, substitution at Lys^7^ (as it is in Lys^7^(PEG_4_-Van)]TP10) or at the C-terminus (as it is in TP10-Ala(PEG_4_-Van) greatly increased the antibacterial activity of the conjugates against MRSA 12673, which was reflected by the MIC values being within the range of those for susceptible bacteria, i.e. ≤1.38 μM. What is more, this effect was more pronounced than that produced by Van-PEG_3_-TP10 and Van-PEG_4_-TP10, where the substitution occurred at the N-terminus. These results are not surprising in light of reports^31,32^ which indicated that for optimal intracellular delivery, TP10 should be connected via the side chain of Lys^7^ to the cargo. Substitution at this site leaves the adjacent basic Lys^18,19^ residues both protonated, which results in stabilization of TP10 C-terminal part, and this in turn is essential to the cell-penetrating process.

On the other hand, the increased antibacterial activity of the conjugates was not as visible in the case of the enterococcal strains. An increase in action was only noticed if *E*. *faecium* was exposed to [Lys^7^(PEG_4_-Van)]TP10 or *E*. *faecalis* to Van-PEG_3_-TP10.

The improvement of antibacterial activity of Van described above may result from versatile interactions between Van and TP10. Both types of interactions, i.e. pharmacodynamic as well as pharmacokinetic, seem to be possible. In order to identify them, it is important to summarize the conjugate’s constituents.

As far as Van is concerned, this antibiotic is a hydrophilic glycopeptide with a molecular weight of 1.45 kDa. Therefore, it poorly penetrates the intracellular compartment. Constitutionally, by inhibiting cell wall synthesis this antibiotic produces a time-dependent bactericidal effect against G+ aerobes and anaerobes. More precisely, Van binds to the D-Ala-D-Ala moiety of the cell wall precursors and, in this way, interferes with the growth of PG, which functions as an exoskeleton that prevents cell rupture due to internal pressure^44^.

TP10, the second constituent of the conjugate, is a short chimeric amphiphatic cationic CPP with an antimicrobial activity (versatile bacterial strains e.g. *S*. *aureus*^25^, *N*. *meningitides*^30^, *E*. *coli*, *P*. *aeruginosa*, *A*. *baumannii*^25^ and fungi e.g. *C*. *albicans*^27^) due to the mastoparan sequence (active antimicrobial peptide)^45^. Moreover, it was found that this CPP possessed potent antibacterial activity against MDR bacteria with high separation rates when isolated from clinics^25^. Similarly, TP10 in this study revealed a comparable action against MRSA clinical strains.

According to recent research, a double-targeted mechanism has been implicated in the TP10’s antibacterial action: (1) damage to the cell lipid envelope by rapid and lethal permeabilization, (2) retardation of genomic DNA migration in the cytoplasm^25^. Both processes are attributable to the peptide’s cationic nature, which is determined by the number of Lys residues (alkaline amino acid) in its molecule. To be specific, due to this quality, TP10 binds preferentially by electrostatic attraction to negatively charged bacterial membranes (high content of phospholipides and lipoteichoic acid – Gram-positive bacteria or lipopolysacharide - Gram-negative bacteria) and phosphate fragments of DNA (polyions)^25^.

With reference to the second mechanism of TP10 antimicrobial action, it was demonstrated that its binding to DNA is a concentration-dependent intrinsic ability of the peptide. It is believed that this mode of antimicrobial action is not affected in known mechanisms of resistance^25^.

In light of the aforementioned features of the conjugate’s components, it may be supposed that the improvement in its antibacterial action reflects a pharmacodynamic interaction between Van and TP10, although it does not meet strictly the definition for synergy against MRSA. Emergence of this kind of interaction is not very surprising due to the fact that each component of the conjugate is targeting different bacterial structures by distinct mechanism(s) of action (Van-inhibition of cell wall synthesis; TP10- permeabilization of cell membrane, binding to the cytoplasmic DNA).

What is more, it appears that thanks to the triple targeted antibacterial action of the conjugates it was possible to overcome Van resistance in MRSA, and, in the case of *E*. *faecium* or *E*. *faecalis*, to diminish the MICs to values characteristic of intermediate resistant strains to Van (5.38–10.77 μM). These bacteria, which are usually multi-drug resistant, are mainly opportunistic and prevalent in hospitals and other health care settings, causing serious life-threatening infections, including endocarditis, meningitis, bacteraemia and osteomyelitis.

Similarly, a covalent approach has been presented by Gomarasca *et al*.^46^ who found that the conjugates of CPPs (Tat - transactivator of transcription derived from HIV, α1 H and α2 H) with gentamacin targeted and effectively killed intracellular pathogenic bacteria such as *Escherichia coli*, *Salmonella enterica serovar Typhimurium*, *Shigella flexneri* if the conjugates were directly applied to infected cells (modified gentamicin protection assay). It is worth stressing that in this case the antibacterial effect of the conjugates was much more evident than that of gentamicin itself. Among them the most prominent action (986-fold reduction of bacteria in comparison to unconjugated gentamicin) was caused by Tat-gentamicin, probably due to the fact that Tat (like TP10 in this study) also possessed a documented antibacterial activity. According to the cited study^46^ the improved antibacterial effect of the CPP-gentamicin conjugates is a result of their effective intracellular penetration. The conclusion does not raise doubts if the action of the conjugates at the level of bacterial cells (functionality assay) is taken into consideration. In this case, the antimicrobial activity of CPP-gentamicin conjugates was almost the same as that after gentamicin treatment.

The next issue of importance in the context of this study is the impact of simple bulk-mixing of Van with TP10 on MRSA strains. A twofold decrease in MIC value in comparison to that of Van was found after exposition of MRSA 6347 to this mixture. There was no effect on MRSA N315 and 12673 strains.

Physical complexation of CPPs with antibiotics gained also attention by others^15,47,48^. The use of this strategy (e.g. Tat + gentamicin) by Gomarasca *et al*.^46^ led to a slightly improved antibacterial effect in comparison to that obtained after gentamicin itself. On the other hand, a much more favourable effect was obtained if mixture of other CPPs, without (P3 and P8) or with (amphiphilic cyclic CPPs) antibacterial activity were used together with Van or tetracycline against MRSA, respectively^15,47^. A drop in MIC values to susceptible levels was characteristic of P3 or P8 mixtures with Van^15^, and a conversion to a bactericidal effect, in turn, was observed if a mixture of an amphiphilic cyclic CPP with tetracycline was used^47^.

The fact that the mixtures of the CPPs and antibiotics produced a various degree of increase in the antibiotic action could result from the assumed strategy itself (physical complexation). It is known that its final products will be depended on the physicochemical properties of the CPP and cargo molecules as well as formulation principles applied. Moreover, a pool of poorly chemically defined compounds varying in size and composition is usually formed after a simple bulk-mixing^22^.

Another aspect of the antimicrobial action of Van-TP10 may concern its possible penetration to the host cells. It is known that Van, due to molecular size and polarity, has poor access to them, and therefore this antibiotic does not effectively kill intracellular bacteria, including MRSA^8^.

On the other hand, TP10 is easily internalized in pro- as well as eukaryotic cells. In the bacteria the internalization takes place *via* direct translocation since endocytosis does not generally occur. In the case of eukaryotic cells it is accepted that both endocytic pathway^22,23,48–51^ and direct translocation^21–23^ are of equal importance in the process of TP10 membrane trafficking. The former one (energy dependent) involves interaction with cell surface PGs leading to endocytic uptake, while the latter one, is a direct membrane translocation (energy independent) which requires permanent or temporary destabilization of the plasma membrane by the peptide’s presence in its lipid layer and may include, e.g. formulation of aqueous transient pores^52,53^ or hydrophilic prepores^54,55^ (both of them with toroidal structure). There are also other potential mechanisms such as scavenger mediated or paracellular translocation which have been proposed for transepithelial permeation^21^. According to many reports, versatile direct mechanisms of membrane translocation and endocytosis may occur simultaneously for most CPPs and this is especially characteristic of the amphiphatic ones^29,45,56–58^. Furthermore, TP10 thanks to the C-terminal of the mastoparan domain may adopt a secondary amphiphatic α-helix structure on the surface of the zwitteronic membranes, which modifies membrane integrity and thereby cellular uptake^33,58^.

Considering the data presented above it is possible to hypothesise that the transporting activity of TP10 enables Van to penetrate not only inside the MRSA cells but also those of the host. A support for such a hypothesis are the results of the experiments with [Lys^7^(PEG_4_-Van)]TP10 carried out on MRSA - infected human cells. As they indicate the conjugate gained access to the cells with a resultant evident bactericidal effect (71% of the intracellular MRSA was killed). At the same time, the action of the conjugate’s components i.e. that of Van or TP10 was much weaker (5% or 26% bacterial killing rate, respectively). Thus, the intracellular bacteria, which are usually inaccessible to this antibiotic may become a target.

Theoretically, the consequences of these processes may be versatile and of significance to Van antimicrobial action. For example, the drug may target the cell wall not only from its outer, but also its inner surface. Since Van has high affinity to D-Ala-D-Ala, it is also possible that this antibiotic interferes with processes at the earlier, cytoplasmic stage of the PG synthesis pathway, i.e. with those which start after ligation of two D-Ala molecules^4,59^.

The next issue which will be considered is the passage of Van-TP10 through BBB. This barrier is a difficult obstacle for a drug to overcome since it possesses unique biological qualities (non-fenestrated brain endothelial cells with tight junctions-TJs) which impose restriction on the transport of unwanted substances into the CNS. Among them is Van with its glycopeptide structure and hydrophilic nature. However, it is intriguing that [Lys^7^(PEG_4_-Van)]TP10 gained access to the brain when investigated in this study on murine cerebral model (*in vivo* experiments). Its amount in the brain was >200 times bigger than that of Van itself (the LC/MS method). In contrast to the conjugate, the co-joint of Van and TP10 did not penetrate the BBB at all (its amount in the brain tissue was undetectable). Thus, TP10 may enlarge the set of CPPs which may be used for trafficking the antimicrobials across the BBB into the CNS, but only if it is used in the conjugated form.

So far, other CPPs^22,52^ have been used for this purpose; examples include Tat and SynB family vectors (derived from natural peptides-protegrines). The former one, as a conjugate with ciprofloxacin indicated an enhanced uptake of the chemotherapeutic into the brain if administered *iv* in rats^60^. The latter CPPs functioned as vehicles for successful brain delivery of doxorubicin, benzylpenicillin and paclitaxel (*in vivo* and *in situ* experiments on mice)^58–60^.

An explanation for the fact that Van-TP10 gained access to the brain is more complex than it appears to be. Generally, the mechanisms responsible for the translocation of the conjugate across the endothelial barrier are likely to share similarities with those exploited for the intracellular delivery of the cargo into other non-cerebral tissues. As has been reported, cargo delivery to them is mostly endocytosis driven and most probably dependent on endosomal escape^35,61^. For the time being it is difficult to predict whether the same mechanisms apply to the transport of the CPP plus cargo across the BBB.

To approach this issue it is worth considering the brain transport of the CPPs. The above mentioned Tat and SynB (arginine rich peptides) penetrated well the BBB with the usage of a non-saturable mechanism, i.e. adsorptive-mediated endocytosis. On the other hand, as this study demonstrated TP10 (lysine reach sequence) did not gain access to the brain. This result is in accordance with those presented by others^62^. The lack of satisfactory concentration of TP10 in the brain (although, mastoparan did)^43^ is presumably due to its low charge density and high rate of efflux. Hence, the presence of Van must have changed the penetrating properties of TP10 if it was used in the form of a conjugate, and this in turn resulted in significant delivery to the brain.

The fact that the cargo has an impact on the process of internalization has been well documented. The most important factors concern cargo nature and characteristics (e.g. origin, chemical structure, size, charge, concentration), formulation approach (covalent or non-covalent), structural and chemical rearrangements (when TP10 as a part of a supramolecular conjugate encounters the plasma membranes), intermolecular interactions between the cargo and CPP^22,52,63–65^. In the case of Van-TP10, a positively charged cargo of small molecular weight (1.45 kDa) was attached covalently to a CPP. Thus, a new entity with different physico-chemical properties was formed which facilitated internalization and access to the brain^64^.

As this study indicated, the formulation approach seems to be of great importance because the use of non-covalent one (simple bulk-mixing of Van with TP10) even worsen the BBB penetrating ability of Van itself. The explanation of this experimental fact is only hypothetical and may involve versatile processes as for example formation of a large complex molecule or antagonistic interaction between Van and TP10 which led to a complete hindrance of the access to the brain compartment.

Since the internalization process is multifactorial and even the cellular trajectories of the same CPP + cargo may differ in distinct cell lines, any indication of the mechanisms involved here seems to be pure speculation. However, it seems to be possible that the BBB transport of Van-TP10 will show similarities to that (adsorptive-mediated endocytosis) indicated for Tat + cargo, SynB vectors + cargo or other CPPs + cargo in cerebral or non-cerebral tissues, respectively^66,67^. Of course, other mechanisms cannot be excluded such as transcellular or paracellular diffusion. With respect to the latter one, the report of M. Kristiansen *et al*.^22^ indicates its contribution to the net delivery of the cargo across the endothelium *via* a direct or indirect effect on the TJs dynamics. Additionally, the efflux processes described for TP10 (mainly P-gp pump)^66^ could be diminished in the case of Van-TP10 usage.

Gaining access to the brain tissue by Van-TP10 (found in this study) together with the propensity of both conjugate’s components to kill certain brain pathogens (e.g. *N*. *meningitidis)* may have important clinical implications. Namely, it would enable its usage in cerebral infections at smaller doses than standard, and this, in turn, would decrease the potential for drug-induced toxicity.

Another interesting point is whether the improved antimicrobial activity and pharmacokinetics of Van-TP10 coincides with elevated cytotoxicity. Such a relationship was not found in this study, despite the fact that TP10, due to its cationic amphipathic nature, is known to be more toxic than other CPPs, but this usually occurs at high concentrations^31,33,35^. Lack of TP10 toxicity was also demonstrated at two different human cell lines (HEK293, HEL299) at this laboratory^34^.

On the other hand, it has been indicated that the presence of cargo decreases the cytotoxicity of the CPPs. In the case of TP10, it occurs especially if coupling is orthogonal at Lys^7^ or Lys^13^ of the sequence^31^. As far as this study is concerned, Van-PEG_3_-TP10 and Van-PEG_4_-TP10 at concentrations equal to those of MIC values for susceptible or intermediately susceptible bacteria, had no hemolytic activity (erythrocytes are used as a model for normal eukaryotic cells). It is remarkable that both compounds in question (chosen on purpose) present N-terminally substituted conjugates of TP10 which are supposed to have higher toxicity than those coupled orthogonally^31^.

Generally, it appears that Van-TP10 conjugates are relatively safe. Perhaps, due to the presence of TP10 in their molecule, they indicate much lower affinity to mammalian cell membranes than to those of the bacteria. The latter ones, being negatively charged, are the main target for the binding of TP10. Furthermore, the opinion presented by El-Andalousi *et al*.^31^ may be adopted here, i.e. the cytotoxic side effects of TP10 + cargo may be decreased compared with the free peptide as a result of the interactions between the constituents of the conjugate, which makes the cellular membranes less exposed to TP10.

In summary, Van as a conjugate with TP10 may constitute a novel treatment modality. It offers an improved clinical response to Van therapy due to an increase in its antimicrobial activity, regardless of whether the infection is localized in the intra-or extracellular compartment, outside or inside the brain. Additionally, as a promising alternative to conventional antibiotics, it may help not only to overcome antimicrobial resistance, but also constitute a defence against the development of multidrug resistance in the bacteria as it would require a complete remodelling of several bacterial structures or the bypassing of main biochemical pathways.

## Conclusions

Conjugation of Van with TP10 improves the antibiotic’s pharmacodynamics (enhanced antibacterial action mainly against clinical strains of MRSA) and pharmacokinetics (significant access to the infected host cells and brain tissue) with no significant increase in toxicity.

## Supplementary information

Supp.Fig.

Supp.Tab.
