# Publisher Correction: Transportan 10 improves the pharmacokinetics and pharmacodynamics of vancomycin

**DOI:** 10.1038/s41598-019-54823-6

**Published:** 2019-11-29

**Authors:** Jarosław Ruczyński, Izabela Rusiecka, Katarzyna Turecka, Agnieszka Kozłowska, Magdalena Alenowicz, Iwona Gągało, Anna Kawiak, Piotr Rekowski, Krzysztof Waleron, Ivan Kocić

**Affiliations:** 1 Faculty of Chemistry, University of Gdansk, Wita Stwosza 63, 80-308 Gdansk, Poland; 2 Department of Pharmacology, Medical University of Gdansk, Debowa 23, 80-204 Gdansk, Poland; 3 Department of Pharmaceutical Microbiology, Faculty of Pharmacy, Medical University of Gdansk, Hallera 107, 80-416 Gdansk, Poland; 4 Department of Biotechnology, Intercollegiate Faculty of Biotechnology, University of Gdansk and Medical University of Gdansk, Abrahama 58, 80-307 Gdansk, Poland

Correction to: *Scientific Reports* 10.1038/s41598-019-40103-w, published online 01 March 2019

The original PDF version of this Article contained an error in Figure 3 which was a duplication of Figure 2. This has now been corrected in the PDF; the HTML version of the paper was correct from the time of publication.

