# Supplementary material for: Transportan 10 improves the pharmacokinetics and pharmacodynamics of vancomycin
Source: Sci Rep. 2019 Mar 1;9:3247. doi: 10.1038/s41598-019-40103-w (PMC6397271; doi:10.1038/s41598-019-40103-w)
Supplement: Supplementary file 1 — Supp.Fig. [file 41598_2019_40103_MOESM1_ESM.pdf]

Transportan 10 improves the pharmacokinetics and pharmacodynamics of vancomycin

Jarosław Ruczyński[2°], Izabela Rusiecka[*1°], Katarzyna Turecka[3], Agnieszka Kozłowska[2], Magdalena Alenowicz[2], Iwona Gągało[1], Anna Kawiak[4], Piotr Rekowski[2], Krzysztof Waleron[3], Ivan Kocić[1]

a

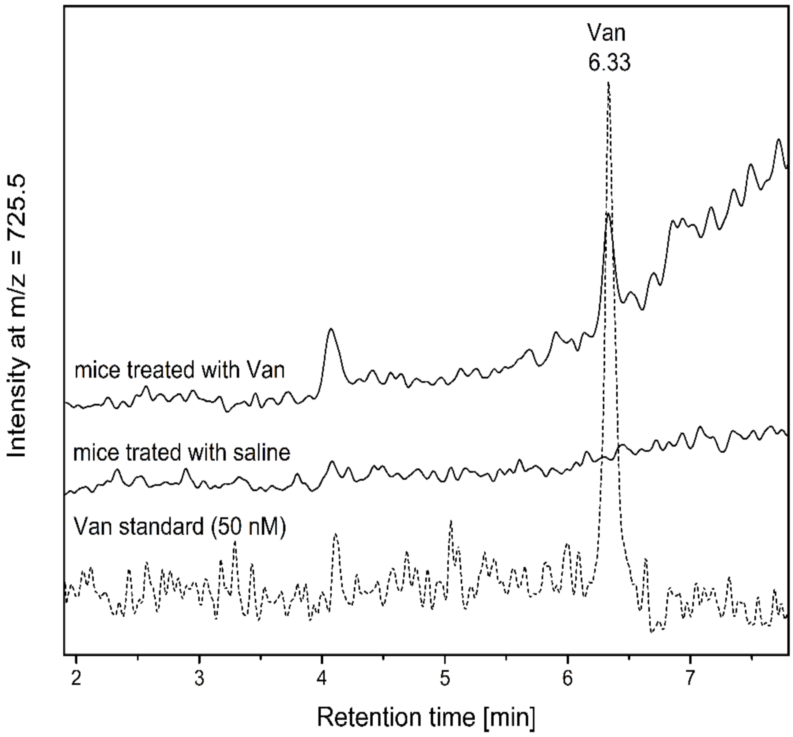

b

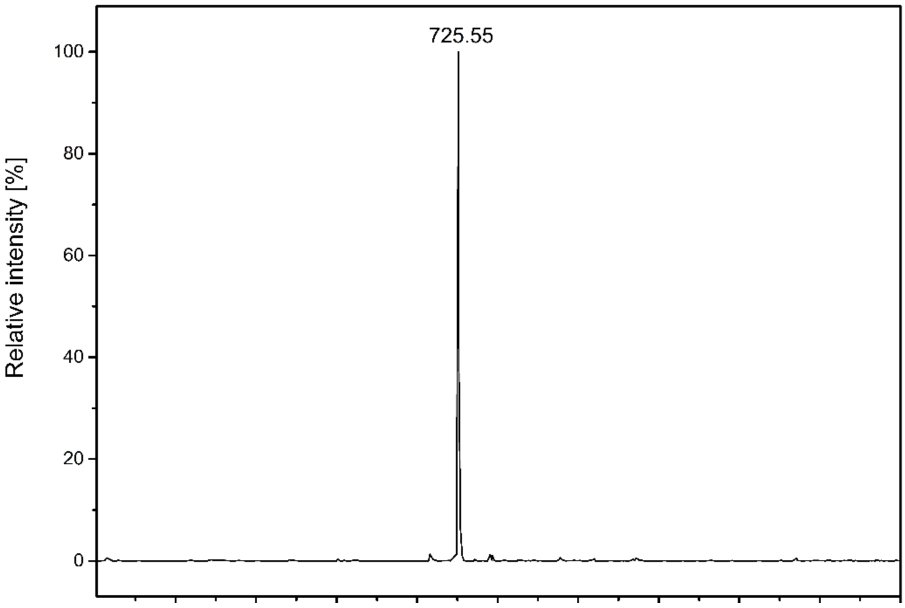

Supplementary Figure 1. Examples of LC-MS chromatograms (a) of brain homogenates from mice treated *iv* with saline and Van (solid lines) and Van standard at a concentration of 50 nM (dashed line), peak at Rt = 6.33 min corresponds to Van, confirmed by peak at m/z = 725.55 $[M+2H]^{2+}$ on ESI-MS spectrum (b).

a

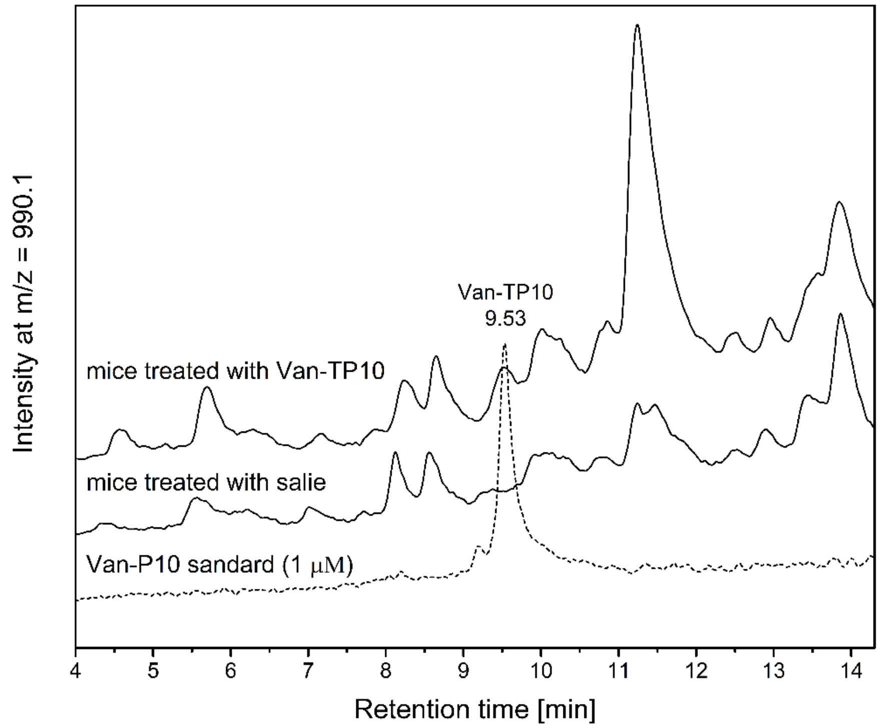

b

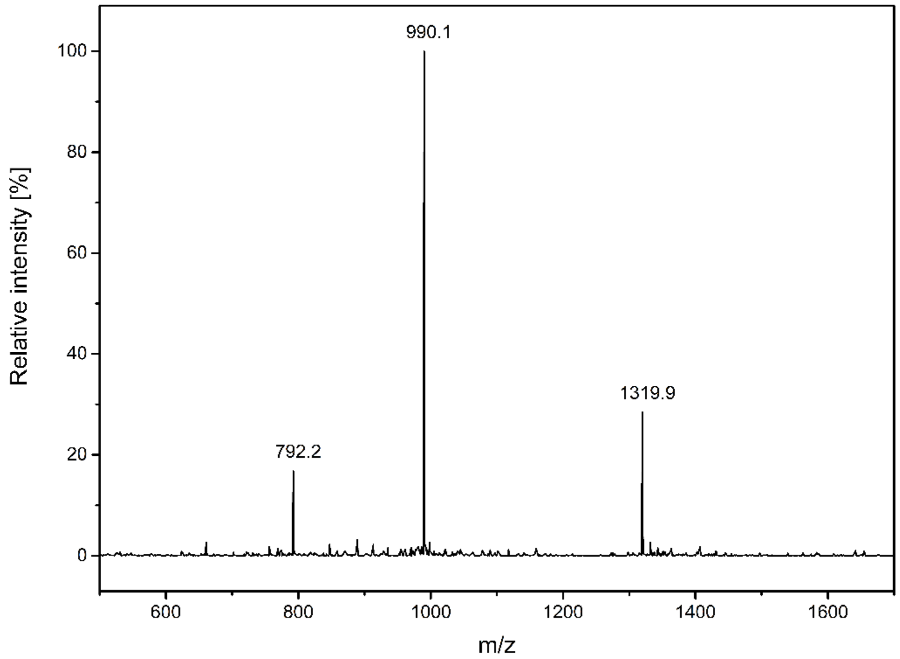

Supplementary Figure 2. Examples of LC-MS chromatograms (a) of brain homogenates from mice treated *iv* with saline and [Lys^7(PEG$_4$-Van)]TP10 (solid lines) and [Lys^7(PEG$_4$-Van)]TP10 standard at a concentration of 1 μM (dashed line), peak at Rt = 9.53 min corresponds to [Lys^7(PEG$_4$-Van)]TP10, confirmed by peak at m/z = 990.10 $[M+4H]^{4+}$ and 1319.90 $[M+3H]^{3+}$ on ESI-MS spectrum (b).

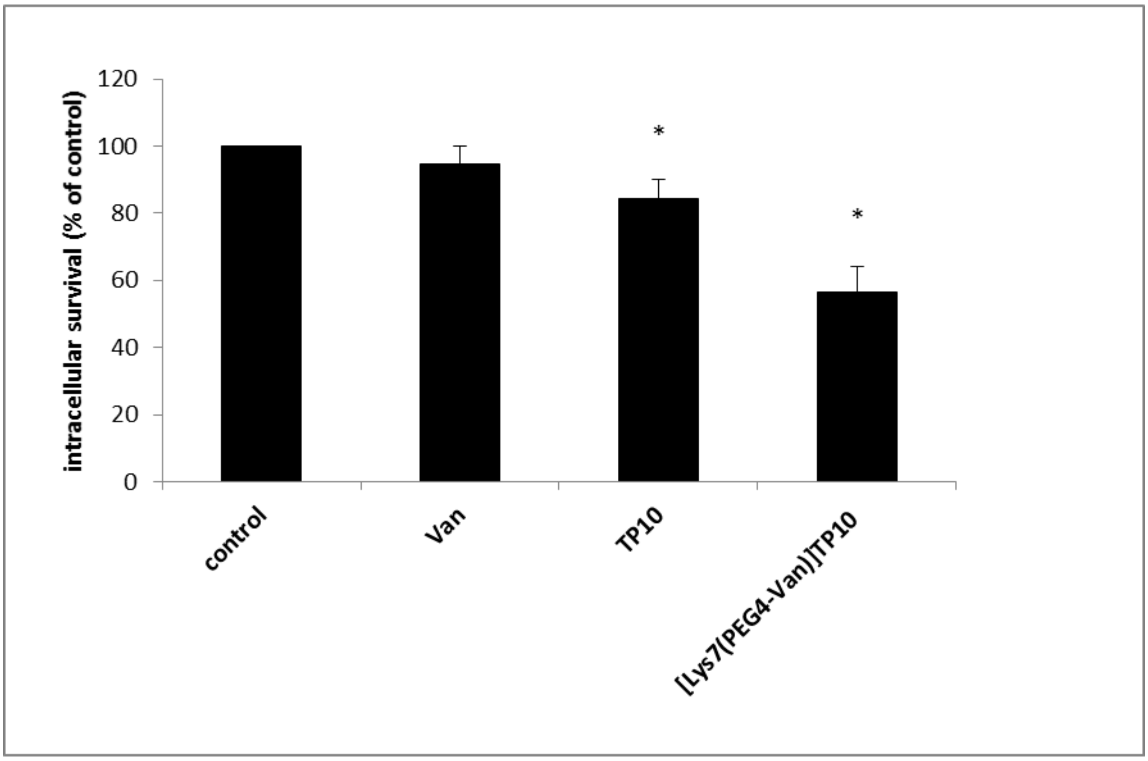

Supplementary Fig. 3. Antimicrobial activity of [Lys^{7}(PEG$_4$-Van)]TP10 against intracellular MRSA h-VISA 6347 strain (8xMIC).
** statistically significant ($p<0.05$) as compared to control (no treatment) and Van*
